# Evaluation of a Brief Intervention for Promoting Mental Health among Employees in Social Enterprises: A Cluster Randomized Controlled Trial

**DOI:** 10.3390/ijerph15102107

**Published:** 2018-09-25

**Authors:** Benedicte Deforche, Jasmine Mommen, Anne Hublet, Winnie De Roover, Nele Huys, Els Clays, Lea Maes, Ilse De Bourdeaudhuij, Jelle Van Cauwenberg

**Affiliations:** 1 Department of Public Health, Ghent University, De Pintelaan 185, 9000 Ghent, Belgium; benedicte.deforche@ugent.be (B.D.); jasmine_mommen@hotmail.com (J.M.); anne.hublet@gmail.com (A.H.); els.clays@ugent.be (E.C.); lea.maes@ugent.be (L.M.); 2 Physical Activity, Nutrition and Health Research Unit, Faculty of Physical Education and Physical Therapy, Vrije Universiteit Brussel, Pleinlaan 2, B-1050 Brussels, Belgium; 3 Vlaams Instituut Gezond Leven (Flemish Institute Healthy Living), Gustave Schildknechtstraat 9, 1020 Brussels, Belgium; info@gezondleven.be; 4 Department of Movement and Sport Sciences, Ghent University, Watersportlaan 2, 9000 Ghent, Belgium; nele.huys@ugent.be (N.H.); ilse.debourdeaudhuij@ugent.be (I.D.B.); 5 Research Foundation–Flanders (FWO), Egmontstraat 5, B-10000 Brussels, Belgium

**Keywords:** workplace health promotion, people with disabilities, resilience, empowerment, coping

## Abstract

Evidence on the effectiveness of workplace mental health promotion for people with disabilities is limited. This study aimed to evaluate the effectiveness of a brief mental health promotion intervention in social enterprises. It had a non-blinded cluster randomized controlled trial design with follow-up one and four months after the intervention. In total 196 employees agreed to participate (86 intervention and 110 control). Empowerment was the main outcome; secondary outcomes were resilience, palliative behavior, determinants of four coping strategies of mental health, quality of life, and life satisfaction. A brief participant satisfaction survey was conducted after the intervention. No significant intervention effect on empowerment was found. However, at one month follow-up, significant favorable effects were found on perceived social support for coping strategies for mental health and on palliative behavior. At four months follow-up, favorable intervention effects were found on quality of life, but unfavorable effects were found on unjustified worrying. In addition, the intervention was well received by the employees. This brief intervention might be a promising first step to improve mental health in people with disabilities working in social enterprises. Nevertheless, additional monitoring by professionals and managers working in the organizations might be needed to maintain these effects.

## 1. Introduction

Health inequalities are an important issue in public health [1]. People with intellectual and physical disabilities experience poorer health than the general population [2,3]. People with disabilities are more likely to experience depression, anxiety, and stress [4,5]. These health inequalities are to a large extent independent of the health impact of disabling impairments and are therefore modifiable through health promoting interventions [6]. According to people with disabilities and researchers, good mental health is a precondition for the performance of other health behaviors (e.g., physical activity, healthy eating, or smoking) and health in general (no health without mental health) [7,8,9]. Therefore, policies and programs promoting mental health among people with disabilities are needed [10]. Existing health promotion programs are often not tailored to the specific needs of people with disabilities and should be adapted [11,12,13,14].

Health promotion, specifically mental health promotion, can contribute to the prevention of depression, anxiety, and stress and enhance quality of life in people with disabilities [11]. Mental health promotion is the process of enhancing the capacity of individuals to take control over their lives and improve their mental health. Mental health promotion generally aims to empower people and to enhance their resilience or ability to recover quickly after confrontation with problems, by increasing their ability to cope with stressful life events [15]. In people with disabilities, teaching them how to recognize when they are feeling stressed and teaching them coping strategies to deal with these difficult experiences are important strategies to promote mental health [16]. Empowerment is often used as primary outcome in mental health promotion programs [17,18,19].

As people with disabilities are often hard to reach, it is important to integrate health promotion in their natural setting, such as their workplace [11]. The Ottawa Charter recognizes the workplace as an important setting for mental health promotion interventions [20]. In Flanders, people with intellectual and physical disabilities have the opportunity to work in social enterprises [21]. Social enterprises employ society’s most fragile members, and in that way, contribute to social cohesion, employment, and the reduction of inequalities. Since April 2015, all social enterprises in Flanders are required to implement a personal development plan for their employees which consists of both work-related and personal goals (e.g., mental health) [22]. Therefore, social enterprises are an ideal setting for mental health promotion in people with disabilities [7]. Workplace mental health promotion can be beneficial for the employee as well as the employer. For example, workplace health promotion increases job satisfaction of the employee; and for the company, health promotion has a positive influence on absenteeism, productivity, and company image [23]. Furthermore, health promotion activities targeting resilience and coping skills increase the employee’s ability to work and meet the demands of their work [24]. Different types of coping have various influences on job-related outcomes. Support seeking coping and positive reframing is associated with an increase in job satisfaction, while avoidant coping is related to a decrease in job satisfaction [25]. Another advantage of workplace health promotion is that intervention sustainability can be facilitated when the intervention is integrated in the company policies [15]. Workplace health promotion also offers the opportunity to provide group sessions rather than individual counseling, which is an advantage for people with disabilities who often depend heavily on their social environment to support them [26]. However, more research on the effectiveness of health promotion programs specifically designed for people with disabilities is needed [12,27]. In order to develop programs addressing the needs of this target population, it is important to take into account their intellectual limitations (e.g., difficulty understanding, lack of knowledge, lack of concentration), psychosocial barriers (e.g., lack of motivation, lack of self-efficacy, poor outcome expectations), and lack of accessibility (e.g., lack of transportation, equipment, money) [7,28,29]. To the best of our knowledge, no prior studies investigated the effects of a workplace mental health promotion intervention tailored to the needs of people with disabilities.

The purpose of this study was to investigate the effects of the intervention “Op weg naar een goed gevoel” (“Towards feeling good”). This intervention is a brief workplace mental health promotion program tailored to the specific needs of people with disabilities and feasible to implement within an organization (e.g., limited amount of group sessions, tools for further follow-up). The intervention is based on an existing intervention, the Goed-gevoel-stoel (“The feel good chair”) from the Flemish Institute Healthy Living and CEDES (center for educational and social projects), an intervention for promoting mental health among disadvantaged groups. This intervention focuses on enhancing empowerment and resilience and aims to improve mental health through the enhancement of protective factors, in accordance with positive psychology [30,31] and the salutogenic model, which is concerned with the relationship between health, stress, and coping [32]. The focus on increasing resilience creates a paradigm shift from focusing on risk factors to the identification of strengths of an individual [33]. More specifically, the intervention aims to increase empowerment by teaching the following coping strategies: searching for help and support, protecting yourself, taking care of yourself, and accepting yourself. These coping strategies are based on several protective factors for mental health [34,35,36,37]. The existing intervention was, however, not yet adapted for implementation within the workplace of people with disabilities and the intervention had not been evaluated through a controlled trial. In this study, the effects on empowerment, resilience, palliative behavior (which is an indicator of coping behavior [38]), determinants of the four coping strategies for mental health (searching for help and support, protecting yourself, taking care of yourself, and accepting yourself), quality of life, and life satisfaction of employees in social enterprises in Flanders (Belgium) was investigated. In addition, participant satisfaction with the intervention was assessed.

## 2. Materials and Methods

For reporting the intervention, study design and analysis, the TIDieR checklist [39] and the CONSORT statement: extension to cluster randomized trials were used [40].

### 2.1. Intervention Description

#### 2.1.1. Development of the Intervention

The Intervention Mapping protocol [41] was used for program development. This protocol guides the development of interventions but can also be used to adapt an existing intervention. First, the existing intervention of the “Goed-gevoel-stoel” (“The feel good chair”) was framed within the Intervention Mapping protocol. The Intervention Mapping protocol consists of several phases [38]. Firstly, the aim and the target group of the existing and adapted intervention were compared. Both interventions had the same aim, but the target group of the existing intervention (disadvantaged groups) differed from the target group of the adapted intervention (employees in social enterprises). Secondly, performance objectives (POs) were prepared based on the objectives of the existing intervention. The same POs were formulated for the adapted intervention. The Attitude, Social influence, and Self-efficacy model (ASE-model) [42] was used as a theoretical framework for the development of the adapted intervention because we found in a previous study using qualitative interviews [7] that these determinants are important in behavior change among people with disabilities. Change objectives (COs) were structured according to the determinants: knowledge, attitude, self-efficacy, and perceived social support. The determinant awareness was added to the theoretical basis of the adapted intervention as self-reflection is also necessary to perform healthy behavior [40]. In other words, prior to aiming for a behavior change among participants, they need to be aware of a risk for themselves [41,43]. The POs, determinants, and COs are presented in Supplementary File 1. Thirdly, several behavioral change techniques (theory-based methods), identified by Bartholomew et al. [41] to be likely to change the identified determinants, were selected. The behavioral change techniques used in the existing intervention were retained for the adapted intervention. Fourthly, these techniques were linked to the program materials (practical strategies) of the existing intervention. The core components (e.g., the four coping strategies) of the existing intervention were also included within the adapted intervention. However, the existing materials were adapted to the needs of employees in social enterprises. These adaptations were based on a previous study [7]. That study demonstrated, for example, the need for an increase in self-efficacy among employees in social enterprises. More specifically, employees in social enterprises reported that they often know which behavior is healthy but the persistence to keep performing the behavior is lacking. Therefore, goal setting was added as a method to the adapted intervention [41] (see Supplementary File 2). The developers of the existing intervention (i.e., Flemish Institute for Healthy Living and CEDES) also provided advice to optimize the materials. The program materials were further adapted through discussion and evaluation of the program by professionals and managers of social enterprises. More specifically, two individual interviews with managers of social enterprises were conducted. Based on these interviews, three adaptations were made. The first adaptation concerned the focus of the sessions. The adapted intervention gave employees the opportunity to talk about problems at work and private problems, while the existing intervention focused on the opportunity to talk about private problems. The opportunity to talk about problems at work was added as the adapted intervention was organized at the workplace. The second adaptation concerned the amount of sessions. The existing intervention consisted of three sessions, while the adapted intervention consisted of two sessions. The content of the sessions remained the same, but the information was spread over two sessions instead of three. The first session of the existing intervention consisted of an assignment where they talked about the difficulties they experienced in their lives (e.g., problems with family). The second session of the existing intervention concerned an assignment about their resilience, where the participants talked about the coping strategies they used in their lives. The last session of the existing intervention consisted of the selection of group activities (e.g., walking in group) in order to perform the four coping strategies in the future. The adapted intervention integrated the assignments about difficulties participants struggle with and resilience within the first session, as separately discussing these items is emotionally stressful for the participants. Only discussing the assignment about difficulties they struggle with would give the participants a negative feeling after the first session. By adding the assignment about their resilience, the first session could be finished positively. This is important within a work setting, because the participants need to resume work after the session. The third adaptation concerned the content of the last session. The last session of the adapted intervention consisted of the preparation of an individual action plan. The employees had to choose individual actions in order to use the four coping strategies when dealing with a problem in the future. We selected individual actions instead of group activities (as used in the existing intervention), as individual actions can be monitored by professionals of social enterprises through the personal development plan of the employee.

#### 2.1.2. Specific Content of the Intervention

The intervention was organized as two group sessions delivered at the organization. Each group consisted of six to ten employees. The intervention was delivered by one member of the research team (JM), to make sure delivery of the program was standardized. In advance, this person followed training about the “Goed-gevoel-stoel” (“The feel good chair”) at the Flemish Institute Healthy Living. To learn and apply the four coping strategies (cf. PO_2_, PO_3_, PO_4_, and PO_5_ in Supplementary File 1), employees had to complete three assignments. In the first session, employees were asked to explain the difficulties they experienced in their lives (e.g., problems with family or stress at work). This first assignment gave them a better understanding of the difficulties they struggle with (cf. theory-based methods in Supplementary File 2, e.g., scenario-based risk information and self-affirmation task). Furthermore, employees could recognize themselves in each other’s stories (cf. theory-based methods in Additional File 2, e.g., provide opportunities for social comparison). The second assignment of the first session gave employees insight into their resilience. For this purpose, a game was used in which several coping strategies were presented on cards in different colors (blue = searching for help and support, red = protecting yourself, green = taking care of yourself, and yellow = accepting yourself). The employees had to choose the coping strategies they used in their lives. Through the chosen colors, they could see which coping strategies they had already, and which strategies were missing (cf. theory-based methods in Supplementary File 2, e.g., active learning and scenario-based risk information). The third assignment was carried out in the second session and consisted of developing an action plan (cf. theory-based methods in Supplementary File 2, e.g., public commitment and goal setting). The employees had to choose individual actions in order to use the four coping strategies when dealing with a problem in the future.

### 2.2. Study Design

**Figure 1:**
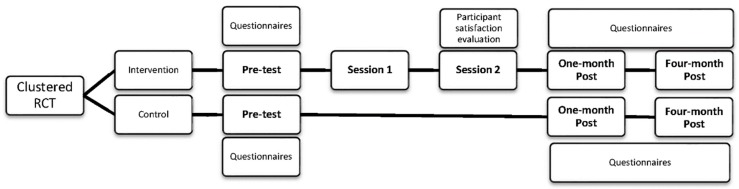
Study design. RCT = randomized controlled trial.

This study had a cluster randomized controlled trial design with a pre-test, an intervention period (two group sessions each lasting four hours and a one-week interval between the two sessions), and two post-tests (one month and four months after the second session) (see Figure 1). The contact information of the organizations was provided by the umbrella-organization for the social economy (CollondSe). From the list of organizations, the largest organizations were randomly selected to be contacted. It was expected that the probability for employees to participate would be higher in large organizations. Moreover, within large organizations there is more variation in gender and age of the employees. The selected organizations were randomly assigned to intervention or control group by a member of the research team (JM). Assignment to the intervention or control group at the individual level was not possible because of the high risk of contamination bias by participants working together in the same organization. Participants from the control group received standard care from the social service of the organization (e.g., safety at work, prevention of back problems, follow-up in case of absence from work, personal conversation if desired, support for financial difficulties, education, training-on-the-job), but did not get any extra psychosocial support. Professionals and managers working in social enterprises invited employees to participate in the study using an invitation provided by the research team. All employees with a basic knowledge of Dutch and employees who wanted to participate and understood the purpose and content of the study (in other words, signed the informed consent) were eligible to participate. The study was approved by the ethical committee of the Ghent University Hospital (2014/1268).

### 2.3. Sample Description and Attrition

**Figure 2:**
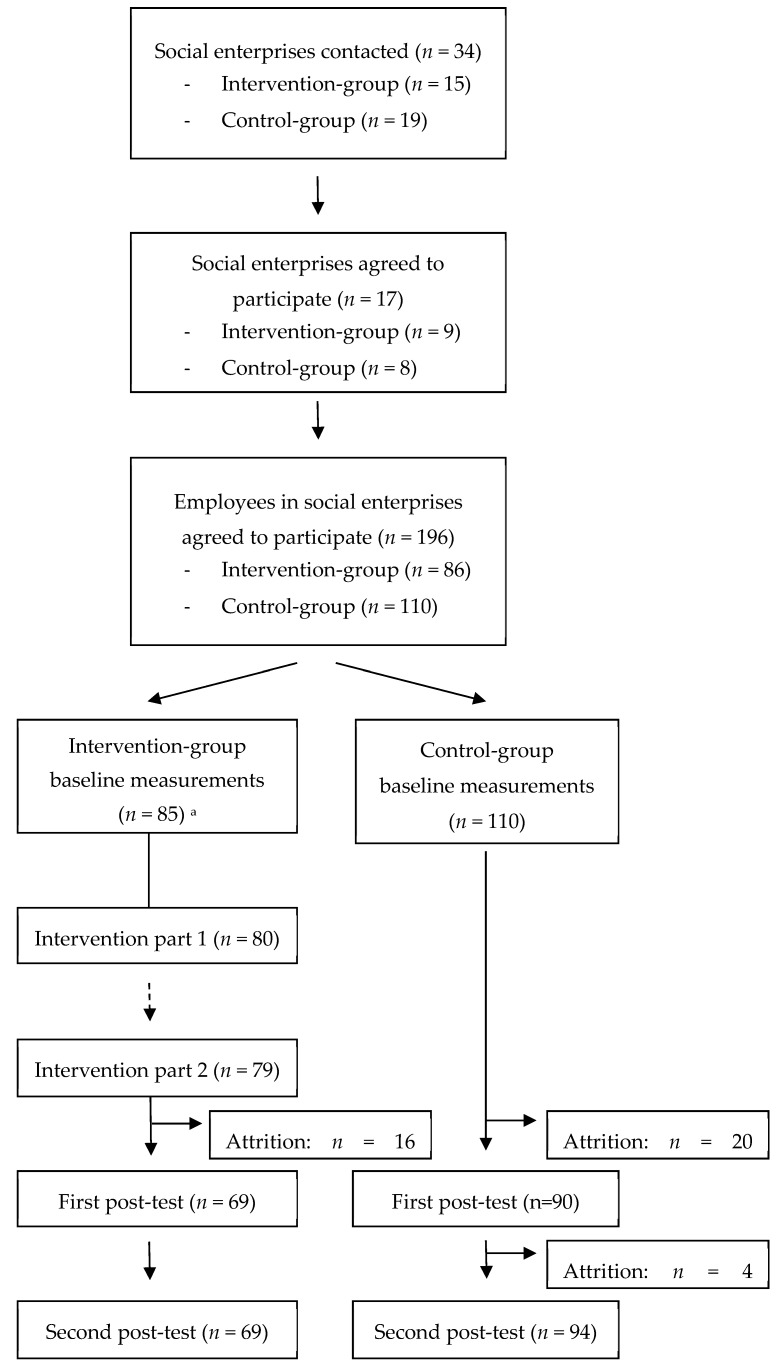
Flow chart of included organizations in the study. ^a^ One participant from the intervention group who originally consented to participate did not complete baseline measurements.

Power analysis was used to calculate the required minimum sample size. In both intervention and control group, 80 employees were needed to be able to detect a 10% increase in the main outcome; empowerment [29] (power = 0.80; alpha = 0.05). Taking into account on average ten employees per organization, initially ten social enterprises per condition (intervention vs. control) were invited to participate in the study. As the minimum sample size determined by the power analysis was not yet reached, extra organizations were contacted. At the end, fifteen organizations for the intervention group and nineteen for the control group were contacted, which resulted in nine participating intervention organizations and eight control organizations. In the intervention group, 86 employees agreed to participate, while in the control group 110 employees agreed to participate. Figure 2 shows the flow chart of the organizations and participants included in the study.

Baseline socio-demographic characteristics of participants are shown in Table 1. The intervention group consisted of 56.5% women and the control group of 52.8% women. The age of the participants varied between 20 and 61 years, from which the largest proportion (36.1% in the intervention group and 33.6% in the control group) was between 41 and 50 years. The majority of the sample went to school until 18 years or older (78.8% in the intervention group and 70.5% in the control group) and followed secondary education for people with disabilities (47.6% in the intervention group and 57.7% in the control group). The majority of the sample was born in Belgium (90.6% in the intervention group and 90.8% in the control group) and had Dutch as mother tongue (81.2% in the intervention group and 86.2% in the control group). Based on chi-square analyses, there were no significant differences in sociodemographic variables between the intervention and control group at baseline.

Attrition rates at the first post-test (one month) were similar in the two groups. At the second post-test (four months), the attrition rate in the control group was 4.3%, while there was no attrition in the intervention group. Attrition was often due to long-term absenteeism, a common problem in social enterprises. To limit attrition, we went back to the organization when the employee had returned (e.g., in case of short-term absenteeism). Another reason for attrition was discontinuation of employment in the organization.

### 2.4. Data Collection Procedures

Data collection for the pre- and post-tests was coordinated by one of the first authors (JM) with assistance of one of the co-authors (NH) (only at post-test) and trained master students (different students at pre- and post-test), who were blind to the condition of the participants at pretest but not at post-test because of the extra ‘satisfaction with the intervention questionnaire’ that was assessed in the intervention group only. JM also implemented the intervention. Pre-testing occurred from January till March 2015. The first post-test was performed one month after the last session and the second post-test four months after the last session. Due to cognitive, language, or reading problems of the target group, the questionnaires of the pre- and post-tests were administered face to face by the trained researchers, but completed by the participants. Participants had unique identifiers to link baseline measurements to the two post-tests. To assess participant satisfaction with the intervention, the intervention group filled out an additional questionnaire with minimal assistance (e.g., opportunity to ask questions) of the trained researchers who were not involved in implementation of the intervention. The researcher who implemented the intervention was not present during this assessment to avoid social desirability bias.

### 2.5. Measures

#### 2.5.1. Sociodemographics

Participants were asked to complete a sociodemographic questionnaire on gender, age, birthplace, mother tongue, age when ending school, and educational level.

#### 2.5.2. Effectiveness Evaluation

##### Empowerment

The Dutch Empowerment Questionnaire [44] was assessed to measure empowerment. Only the self-management scale (alpha: 0.74) was questioned in order to limit the number of items for the participants. The self-management scale includes items which reflect the four coping strategies of our intervention. The psychometric properties of the Dutch Empowerment Questionnaire were studied in people with psychiatric problems and the concurrent validity was found to be acceptable [44].

##### Resilience

Resilience was assessed by four items from the Resilience Response scale [45] which were first adapted to the literacy level of the target group in a preliminary study [46]. The first item was: If you are sad or angry, how difficult is it to get out of your bed, to eat, to work or to shop (resilience daily activities)? The second item: If you are sad or angry, does it take a lot of time to feel well again (duration mental recovery)? The third item: If you are sad or angry, do you know that it will get better and it will be fine (optimism)? The fourth item: How often do you think that something will go wrong (e.g., at work, at home, or in my relationship) (unjustified worrying). The test-retest reliability of the items has been found acceptable (0.63 to 0.77) among employees in social enterprises [46]. For this study, the original response category (Yes/No) was replaced by a 5-point Likert-scale (never, seldom, sometimes, often, and always) to allow more variation in the responses.

##### Palliative Behavior

Palliative behavior of the employees, which is an indicator of coping style, was questioned by the P3 (Portzky’s palliative pallet scale) [38]. The concept palliative behavior was first mentioned in the Utrechtse Coping list [47] and reflects stress reducing activities. A distinction is made between positive activities (e.g., walking) and destructive activities (e.g., smoking). From the P3, subscales can be calculated (e.g., the subscale suicide is the sum score of the items thinking about death and suicidal thoughts) [38]. The test-retest reliability of the items has been reported to be acceptable (0.81 for positive activities and 0.71 for destructive activities). Furthermore, convergent validity of the P3 and the NEO-FFI-3 personality questionnaire was examined and a correlation (r = 0.33) was found with the subscale Extraversion [48]. The criterion validity was also studied. The presence of destructive activities was found to be a predictor for a suicide attempt. In addition, the P3 questionnaire is very suitable to detect effects at short term as it questions actions (e.g., walking) on which change can be expected after one month [38].

Sum scores were calculated for the destructive activities and suicidal ideations (sum score of the items thinking about death and suicidal thoughts). The scale positive palliative behavior could not be calculated in the present study because two items of the positive palliative behavior were not included (sexual activities without partner and sexual activities with partner). These items were considered to be too private to communicate to professionals and managers of social enterprises. Nevertheless, the scale destructive palliative behavior and suicidal ideations are relevant indicators of the palliative behavior of the person [38].

##### Determinants of Coping Strategies

Changes in the determinants (attitude, self-efficacy, and perceived social support) of the four coping strategies (searching for help and support, protecting yourself, taking care of yourself, and accepting yourself) were assessed through a self-developed questionnaire based on the ASE-model [42]. For example, the attitude related to the coping strategy “protecting yourself” was measured by the item: I find that protecting myself (e.g., stand up for myself) makes me feel better. Self-efficacy related to this coping strategy was measured by the item: I find it difficult to protect myself (e.g., stand up for myself). Perceived social support linked to this coping strategy was questioned by the item: People around me (e.g., family, friends or colleagues) understand if I protect myself (e.g., stand up for myself). Each item was measured on a 5-point Likert-scale (strongly disagree, disagree, neutral, agree, and strongly agree). Determinants of the three other coping strategies (searching for help and support, taking care of yourself, and accepting yourself) were assessed similarly. Negative items were recoded, and sum scores were calculated for each determinant. Internal consistency of each determinant was examined with Cronbach Alpha reliability coefficients (alpha for attitude: 0.58, alpha for self-efficacy: 0.71, and alpha for perceived social support: 0.77).

##### Quality of Life and Life Satisfaction

Quality of life was measured with the EQ-5D-5L. This questionnaire consisted of two parts: the descriptive system and the EQ visual Analogue scale (EQ VAS). The descriptive system comprised five dimensions (mobility, self-care, usual activities, pain/discomfort, anxiety/depression) and was measured on a 5-point Likert-scale (no problems, slight problems, moderate problems, severe problems, and extreme problems). Index values for the EQ-5D-5L dimension scores were calculated with the EQ-5D-5L Crosswalk Index Value Calculator (an algorithm) from which one total score representing quality of life was calculated [49]. The EQ VAS is a rating scale with end points of 0 to 100 to be used as a visual aid to measure health [50] and was used as an indicator of life satisfaction (physical health). The EQ-5D-5L has recently been found to be valid in a diverse patient population in six countries [51].

Life satisfaction (mental health) was questioned with the Cantril ladder [52]. The test-retest reliability of the Cantril ladder has been found acceptable (0.76) among employees in social enterprises [46].

It should be noted that for all outcome measures higher scores represented more favorable levels of the outcome measures, except for duration of mental recovery, unjustified worrying, destructive activities, and suicidal ideations.

##### Participants’ Satisfaction with the Intervention

To assess participant satisfaction with the intervention, a self-developed questionnaire was used in which employees could evaluate the content of the intervention, the instructor, and the duration of the intervention. This questionnaire was based on the participant satisfaction questionnaire of the evaluation of the Goed-gevoel-stoel (“The feel good chair”) from Flemish Institute Healthy Living. Each item was measured on a 5-point Likert-scale (strongly disagree, disagree, neutral, agree, and strongly agree). Only the item ‘I found the number of sessions (namely two)’ had three response categories: too long, ideal, or too short.

### 2.6. Data Analysis

Data analysis was performed using R version 3.3.1. In order to evaluate intervention effects, multilevel general linear regression analyses (three levels: repeated measures–participants–social enterprises) were fitted using the lme4-package. This implies that group allocation was specified at the social enterprise-level and that all participants completing baseline measurements were included in the statistical model. All outcome measures pertain to the participant-level. Intraclass correlation coefficients (ICC) were calculated from an intercept-only model to estimate the proportion of variance explained by differences between social enterprises. Intervention effects were examined by estimating the interaction effects between group (intervention versus control) and time (one and four months versus baseline). We calculated the percentage of overall variance in the outcome explained by the full model (including main effects of group and time and their interaction effect). Additionally, the overall explained variance by significant interaction effects was calculated. Significant interaction effects were probed by estimating the time effects in the intervention and control group separately and graphs were created for illustration. All analyses were performed separately for each outcome measure. The level of significance was set at 0.05 for main effects and 0.10 for interaction effects [53].

Participant satisfaction with the intervention was examined by calculating the percentages of intervention participants (strongly) agreeing with the statements concerning satisfaction with the content of the intervention, the instructor, and the duration. 

## 3. Results

### 3.1. Effectiveness Evaluation

Means and standard deviations of the outcome variables in the intervention and control group at baseline, one month and four months of follow-up are presented in Table 2.

**Figure 3:**
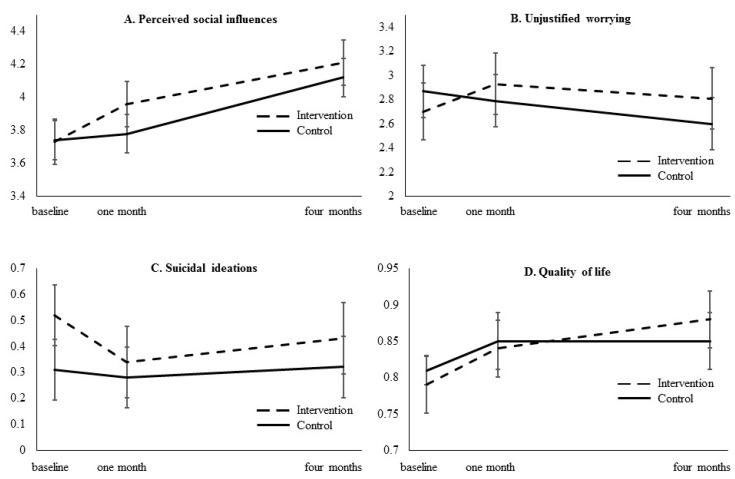
Significant intervention effects.

Results obtained from the multilevel general linear regression analyses for the intervention effects are shown in Table 3. Significant unfavorable intervention effects on unjustified worrying (resilience) were observed at one and four months. Significant favorable intervention effects were observed for suicidal ideations and perceived social support towards coping strategies at one month of follow-up and for quality of life at four months of follow-up. These intervention effects are presented graphically in Figure 3.

For perceived social influences and quality of life, higher scores represented more favorable levels; for unjustified worrying and suicidal ideations, higher scores represented less favorable levels.

For unjustified worrying (resilience) in the control group, no significant change from baseline to one month was observed (b = −0.09, SE = 0.11, *p* = 0.44) and a significant decrease was observed from baseline to four months of follow-up (b = −0.27, SE = 0.11, *p* = 0.01). However, in the intervention group, no significant changes in unjustified worrying were observed from baseline to one month (b = 0.23, SE = 0.13, *p* = 0.08), nor to four months of follow-up (b = 0.11, SE = 0.13, *p* = 0.40). The interaction effect between group and time explained 0.27% of the variance in unjustified worrying.

For suicidal ideations (coping), no significant changes were observed between baseline and one month (b = −0.03, SE = 0.05, *p* = 0.56) and four months of follow-up (b = 0.01, SE = 0.05, *p* = 0.90) in the control group. In the intervention group, a significant decrease in suicidal ideations was observed between baseline and one month (b = −0.19, SE = 0.06, *p* = 0.002), but no significant change was observed at four months of follow-up (b = −0.09, SE = 0.06, *p* = 0.15). The interaction effect between group and time explained 0.17% of the variance in suicidal ideations. 

For perceived social support towards coping strategies, no significant change was observed between baseline and one month of follow-up in the control group (b = 0.04, SE = 0.07, *p* = 0.59), whereas a significant increase was observed in the intervention group (b = 0.23, SE = 0.08, *p* = 0.004). From baseline to four months of follow-up, a significant increase in perceived social support was observed both in the control group (b = 0.38, SE = 0.07, *p* < 0.001) and intervention group (b = 0.47, SE = 0.08, *p* < 0.001). The interaction effect between group and time explained 0.23% of the variance in perceived social support.

For quality of life, significant increases from baseline to one month (control: b = 0.04, SE = 0.01, *p* = 0.01; intervention: b = 0.05, SE = 0.02, *p* = 0.003) and four months of follow-up (control: b = 0.04, SE = 0.01, *p* = 0.01; intervention: b = 0.09, SE = 0.02, *p* < 0.001) were observed in the control and intervention group, but the increase to four months of follow-up was significantly stronger in the intervention compared to the control group. The interaction effect between group and time explained 0.12% of the variance in quality of life.

No significant intervention effects were found on empowerment, resilience daily activities (resilience), duration mental recovery (resilience), optimism (resilience), destructive activities (coping), attitude, and self-efficacy towards coping strategies and life satisfaction (mental and physical health).

### 3.2. Participant Satisfaction with the Intervention

The results of the assessment of participant satisfaction are shown in Table 4. The majority of participants found the assignment about difficulties they struggle with (94.8%) and resilience (94.7%) useful and it gave them a better understanding of the difficulties they struggle with (88.3%) and resilience (90.8%). Most participants found the actions, from which they could choose during session two, useful (97.4%). These actions gave them also good ideas about the implementation of the four coping strategies for mental health (93.4%). Furthermore, the majority of participants reported to be motivated to perform the actions (88.3%) and all participants reported that they understood the meaning of the four colors (100.0%). In addition, most participants felt understood by the instructor (93.4%) and all participants found that the instructor gave a clear explanation of the assignments (100.0%). The majority said that the sessions provided a good feeling (89.6%) and that they felt good in the group (98.7%). Most participants (73.3%) also found the number of sessions (two) ideal, 21.3% found two sessions too short, while only 5.3% reported it to be too long.

## 4. Discussion

The aim of this study was to examine the effectiveness of a brief mental health promotion intervention “Op weg naar een goed gevoel” (“Towards feeling good”) for employees in social enterprises. Furthermore, participant satisfaction with the intervention was assessed. It was expected that the intervention would increase empowerment, resilience, coping behavior, life satisfaction, and quality of life. Furthermore, the intervention aimed to influence the determinants (attitude, self-efficacy, and perceived social support) of the coping strategies of mental health. The effects at short term (one month after the intervention) and at four months follow-up were studied.

At follow-up, a small but significant decrease in unjustified worrying (resilience) was seen in the control group, while no change was found in the intervention group. The decrease in unjustified worrying in the control group was unexpected and we have no clear explanation for this observation. It might be that the intervention group was more aware of the difficulties they struggle with by following the program, resulting in a slightly higher score on unjustified worrying at follow-up compared to the control group. We tried to anticipate such unfavorable intervention effects by combining the first two assignments in the first session, but a more extensive program (including more than two sessions) might be needed to prevent unfavorable effects on unjustified worrying. Preventing an increase in unjustified worrying is crucial, as there is a link between worrying and various mental health outcomes such as stress, depression, and anxiety [14,54]. Future intervention studies could apply a mixed-methods approach to obtain qualitative insights into the mechanisms behind (un)anticipated intervention effects. In contrast to the unfavorable effect on unjustified worrying, a small but significant positive short-term intervention effect was found on suicidal ideations, but this was no longer significant at follow-up.

At short term, a small but significant positive intervention effect was found for perceived social support towards coping strategies. This short-term intervention effect is probably due to the fact that the intervention consisted of group sessions which gave the employees the opportunity to support each other during the sessions. An increase in perceived social support at short term is very positive because social support can increase resilience, which in turn has a positive effect on mental health [55]. However, at follow-up we found a small increase in social support both in the intervention and control group. This short-term positive effect on social support may have been lost because participants were working on their individual actions without discussing these with each other. Maybe the initial positive effect could have been maintained by a more extensive program using extra strategies encouraging employees to communicate with each other about their actions and to support each other [41]. Professionals and managers working in social enterprises could organize, for example, an extra group session where employees can talk about the difficulties they have experienced in performing their actions. In this way, employees could support and help each other. At four months follow-up, we observed a very small but significant positive intervention effect on quality of life. It was expected that the intervention would only have an impact on this variable in the longer term, because quality of life is difficult to change in the short term [41].

No significant intervention effect on empowerment, the main outcome of the intervention, was found. In our study population, participants had relatively high baseline values on empowerment (3.82 ± 0.47), compared to average norm scores (3.66 ± 0.67) [44]. Employees with high baseline values were probably more willing to participate, because they were motivated to work on their health [56]. The sample may also have been biased due to the fact that we used a nonprobability sampling at the individual level [57]. Due to these high baseline values, a significant increase in empowerment as a result of the intervention was difficult to obtain. In addition, the study was somewhat underpowered. Although our total sample size was sufficient at pre-test (86 participants in the intervention group and 110 in the control group), our intervention group consisted of 69 instead of the anticipated 80 participants due to attrition at one month and four months follow-up. Attrition, which is a common problem in intervention studies [58], may explain the absence of a significant effect on empowerment. Future studies could apply over-recruitment or provide participants with incentives to ensure that sufficient participants complete the intervention. As empowerment can be an outcome of a mental health promotion program, as well as a process towards mental health promotion [59], it might be interesting for future studies to investigate empowerment as a potential mediator or moderator of intervention effects [60].

From the effectiveness evaluation, we can conclude that the intervention “Op weg naar een goed gevoel” (“Towards feeling good”) has some positive effects on the mental health of employees in social enterprises particularly in the short term, but this program—with its two sessions of four hours each—may not have been intensive enough to maintain these effects in the longer term. The READY program for instance, a group resilience training program at work to promote psychosocial wellbeing in adults, contains eleven sessions of two hours each and showed significant favorable intervention effects after three months [61]. The current intervention fits within the contemporary mental health promotion practice to increase people’s individual resources and psychological strengths. In line with the positive psychology tradition in public health, the ultimate aim is to enhance quality of life by developing personal resilience skills [62]. Workplaces are considered as crucial settings to deliver mental health promotion, and while programs merely focusing on stress management remain a dominant strategy, a more integrated approach aiming at increased well-being and better mental health is called for [63]. A broad range of possible interventions for mental health promotion at the workplace have been described and it is difficult to draw firm conclusions on their effectiveness. However, several systematic reviews pointed out that workplace interventions particularly aimed at mental health improvement induce effects of usually moderate or small effect size, or generally do not succeed in improving all targeted outcomes [64,65]. It should be stressed though, that from a public health perspective, interventions of modest effect size may be cost-effective [64].

To maintain the short-term effects of the current intervention in the longer term, additional monitoring by professionals and managers working in the organizations might be needed. Follow-up is essential after the preparation of an action plan because the transformation of an intention into behavior is difficult [66]. Follow-up must be integrated within policies and functions of the organizations. The individual actions of the employee, prepared during the last session of the intervention, could be included within a personal development plan. All social enterprises in Flanders are required to implement a personal development plan for their employees, which consists of both work-related and personal goals (e.g., mental health) [23]. From the participant satisfaction survey, we can conclude that the intervention was positively received by the employees. They found the assignments useful, felt understood by the instructor, and felt good in the group. This may be due to the fact that the intervention was tailored to participants’ needs and possibilities [65]. The majority of participants also found the number of sessions (two) perfect.

There are some limitations of the study that need to be acknowledged. First, due to intellectual problems, language problems, or reading problems, researchers who were not blind to the condition of the participants administered the questionnaires face to face; this may have led to social desirability bias and/or Rosenthal effects [59]. This was prevented by emphasizing that there were no right or wrong answers and by encouraging participants to be honest. Second, all measurements were based on self-report, which might not reflect the actual level of employees’ mental health. Third, the positive formulation of all items assessing participant satisfaction with the intervention may have biased their answers. In addition, temporary personal factors (e.g., tiredness or an argument with friends or family) may also affect measurements of mental health [67]. Next, participants from the intervention and control group may have relied upon standard assistance from the social service of the organization during the study period, but this was unfortunately not monitored and could therefore not be controlled for in the analyses. Finally, despite the importance of multilevel interventions [41], the current intervention was individually based and did not target the workplace environment.

Despite these limitations, we want to emphasize that this is the first study investigating the effects of a workplace mental health promotion intervention in people with disabilities. Promoting mental health of disadvantaged people can reduce social inequalities in mental health [10]. The emphasis of the intervention was on the strengths of employees instead of focusing on risk factors. Such a strengths-based psychological climate has positive implications for employees’ mental health [68] and reduces the stigma on mental illness [69]. The current overall trend in mental health promotion is to enhance capacities and build resilience and well-being through supportive environments. Specifically for the workplace, interventions focus mainly on providing supportive environments aiming to reduce psychosocial stressors and increase coping resources [63].

## 5. Conclusions

The mental health promotion intervention “Op weg naar een goed gevoel” (“Towards feeling good”) showed some positive effects at both short term and at four months follow-up. However, no significant intervention effect on empowerment, the main outcome, was found. This was possible due to the relatively high baseline values of our participants on empowerment. Therefore, it is recommended to implement the intervention within a sample with lower baseline values on empowerment. This brief intervention might be a promising first step to improve mental health in people with disabilities working in social enterprises. Nevertheless, additional monitoring by professionals and managers working in the organizations might be needed to maintain the effects.

## Figures and Tables

**Table 1:** Sample characteristics.

Variable	Intervention Group (*n* = 86)	Control Group (*n* = 108)	*X* ^2^	*p*
Gender (% women)	56.5	52.8	0.26	0.61
Age categories (%)			0.26	0.97
20–30 years	16.9	15.9		
31–40 years	21.7	24.3		
41–50 years	36.1	33.6		
51–61 years	25.3	26.2		
Age when ending school (% 18 years or older)	78.8	70.5	1.71	0.19
Educational level (%)			1.18	0.56
Secondary education for people with disabilities	47.6	57.7		
Vocational or technical secondary education	41.3	32.7		
General secondary or tertiary education	11.1	9.6		
Birthplace (% other than Belgium)	9.4	9.2	0.003	0.96
Mother tongue (%)			4.01	0.13
Dutch	81.2	86.2		
Dutch and other	16.5	8.3		
Other	2.4	5.5		

**Table 2:** Outcome variables in the intervention and control group at baseline, one month, and four months.

Outcome	Group	Baseline (M ± sd)	One Month (M ± sd)	Four Months (M ± sd)
Empowerment	Intervention	3.8 ± 0.4	3.9 ± 0.4	4.0 ± 0.5
	Control	3.8 ± 0.5	3.9 ± 0.5	4.0 ± 0.6
Resilience daily activities	Intervention	3.5 ± 1.1	3.7 ± 1.2	3.9 ± 1.2
	Control	3.2 ± 1.2	3.4 ± 1.1	3.5 ± 1.3
Duration mental recovery	Intervention	2.6 ± 1.1	2.5 ± 1.2	2.8 ± 1.1
	Control	2.3 ± 1.2	2.3 ± 1.1	2.3 ± 1.2
Optimism	Intervention	3.7 ± 1.0	3.8 ± 0.9	4.2 ± 0.9
	Control	3.8 ± 1.1	3.9 ± 1.0	4.0 ± 1.0
Unjustified worrying	Intervention	2.7 ± 1.1	2.9 ± 1.1	2.8 ± 1.2
	Control	2.9 ± 1.0	2.8 ± 1.0	2.6 ± 1.0
Destructive activities	Intervention	0.7 ± 0.3	0.6 ± 0.3	0.7 ± 0.3
	Control	0.7 ± 0.3	0.7 ± 0.2	0.7 ± 0.3
Suicidal ideations	Intervention	0.5 ± 0.7	0.3 ± 0.4	0.4 ± 0.6
	Control	0.3 ± 0.6	0.3 ± 0.5	0.3 ± 0.5
Attitude	Intervention	3.9 ± 0.5	4.0 ± 0.5	4.2 ± 0.6
	Control	3.9 ± 0.5	3.9 ± 0.5	4.1 ± 0.6
Self-efficacy	Intervention	3.2 ± 0.7	3.2 ± 0.8	3.6 ± 0.9
	Control	3.4 ± 0.7	3.5 ± 0.7	3.7 ± 0.9
Perceived social support	Intervention	3.7 ± 0.5	4.0 ± 0.6	4.2 ± 0.7
	Control	3.7 ± 0.6	3.7 ± 0.6	4.1 ± 0.6
Quality of life	Intervention	0.79 ± 0.17	0.84 ± 0.15	0.88 ± 0.13
	Control	0.81 ± 0.15	0.85 ± 0.15	0.85 ± 0.16
Life satisfaction (physical health)	Intervention	66.9 ± 20.6	69.2 ± 20.9	72.0 ± 18.8
	Control	67.1 ± 19.6	73.3 ± 19.1	74.7 ± 19.7
Life satisfaction (mental health)	Intervention	7.4 ± 2.5	7.8 ± 2.5	7.2 ± 2.5
	Control	7.7 ± 2.6	7.8 ± 2.6	7.7 ± 2.4

M = mean, sd = standard deviation. For all outcome measures higher scores represented more favorable levels of the outcome measures, except for duration mental recovery, unjustified worrying, destructive activities, and suicidal ideations. For the control group, the number of valid measures ranged from 108 to 110, 93 to 93, and 92 to 95 across variables at baseline, one, and four months, respectively. For the intervention group, the number of valid measures ranged from 83 to 85, 68 to 70, and 66 to 68 across variables at baseline, one, and four months, respectively.

**Table 3:** Intervention effects at one and four months.

Outcomes	ICC	Intercept	Group (ref. = Control)		Time 1 (One Month)		Time 2 (Four Months)		Group * Time 1		Group * Time 2		% Variance Explained ^a^
		b (SE)	b (SE)	*p*	b (SE)	*p*	b (SE)	*p*	b (SE)	*p*	b (SE)	*p*	
Empowerment	0.04	3.86 (0.06)	−0.04 (0.09)	0.65	0.11 (0.05)	**0.03**	0.22 (0.05)	**<0.001**	−0.02 (0.08)	0.75	0.01 (0.08)	0.87	2.10
Resilience daily activities	0.01	3.17 (0.11)	0.32 (0.17)	0.06	0.18 (0.13)	0.16	0.33 (0.13)	**0.01**	−0.01 (0.17)	0.97	0.11 (0.20)	0.58	4.00
Duration mental recovery	0.08	2.32 (0.15)	0.30 (0.22)	0.17	−0.06 (0.12)	0.61	0.02 (0.12)	0.86	−0.08 (0.18)	0.66	0.10 (0.18)	0.58	1.77
Optimism	0.00	3.75 (0.09)	−0.06 (0.14)	0.65	0.09 (0.11)	0.44	0.23 (0.11)	**0.04**	−0.01 (0.17)	0.94	0.26 (0.18)	0.14	1.81
Unjustified worrying	0.00	2.87 (0.11)	−0.17 (0.16)	0.30	−0.09 (0.11)	0.44	−0.27 (0.11)	**0.01**	0.31 (0.17)	**0.07**	0.38 (0.17)	**0.03**	0.00
Destructive activities	0.00	0.72 (0.03)	−0.01 (0.04)	0.77	−0.004 (0.03)	0.88	−0.04 (0.03)	0.10	−0.06 (0.04)	0.11	0.002 (0.04)	0.96	0.00
Suicidal ideations	0.02	0.31 (0.06)	0.21 (0.09)	**0.01**	−0.03 (0.05)	0.56	0.01 (0.05)	0.90	−0.15 (0.08)	**0.05**	−0.09 (0.08)	0.24	1.71
Attitude	0.01	3.87 (0.06)	0.05 (0.09)	0.59	0.08 (0.06)	0.20	0.23 (0.06)	**<0.001**	0.01 (0.09)	0.95	0.05 (0.09)	0.59	1.00
Self−efficacy	0.17	3.42 (0.13)	−0.26 (0.19)	0.16	0.13 (0.08)	0.12	0.29 (0.08)	**0.001**	−0.09 (0.13)	0.47	0.13 (0.13)	0.31	4.42
Perceived social support	0.00	3.74 (0.06)	−0.01 (0.09)	0.93	0.04 (0.07)	0.59	0.38 (0.07)	**<0.001**	0.19 (0.10)	**0.06**	0.10 (0.10)	0.36	3.40
Quality of life	0.00	0.81 (0.01)	−0.03 (0.02)	0.23	0.04 (0.01)	**0.01**	0.04 (0.01)	**0.01**	0.01 (0.02)	0.53	0.05 (0.02)	**0.02**	1.91
Life satisfaction (physical)	0.00	67.14 (1.88)	−0.20 (2.86)	0.95	7.08 (2.03)	**0.001**	7.88 (2.01)	**<0.001**	−4.63 (3.11)	0.14	−1.80 (3.13)	0.57	1.40
Life satisfaction (mental)	0.00	7.73 (0.24)	−0.30 (0.36)	0.40	0.02 (0.30)	0.94	−0.02 (0.30)	0.96	0.37 (0.46)	0.42	−0.12 (0.46)	0.79	0.00

ICC = intraclass correlation coefficient, Ref. = reference category, b = multilevel linear regression estimate, SE = standard error. Bold *p*-values indicate significant main effects (*p* < 0.05) or interaction effects (*p* < 0.10). For all outcome measures higher scores represented more favorable levels of the outcome measures, except for duration mental recovery, unjustified worrying, destructive activities, and suicidal ideations. ^a^ percentage of overall variance in the outcome explained by the full model (including main effects of group and time and their interaction effect).

**Table 4:** Participants’ satisfaction with the intervention.

Questions	Percentages		
	(Strongly) Agree	I Don’t Know	(Strongly) Disagree
I found the assignment about burden useful (*n* = 77) ^1^	94.8	3.9	1.3
Through session 1, I have more understanding about my own burden (*n* = 77)	88.3	3.9	2.6
I found the assignment about resilience useful (*n* = 75)	94.7	4.0	1.3
Through session 1, I have more understanding about my own resilience (*n* = 76)	90.8	6.6	2.6
I found the proposed actions useful (*n* = 77)	97.4	1.3	1.3
I understand the four colors (*n* = 77)	100.0	0.0	0.0
The proposed actions give good ideas about my life (*n* = 76)	93.4	1.3	5.2
I am motivated to perform the actions (*n* = 77)	88.3	6.5	5.2
The sessions caused a good feeling (*n* = 77)	89.6	10.4	0.0
I felt understood by the teacher (*n* = 77)	93.5	5.2	1.3
The teacher gave a clear explanation of the assignments (*n* = 77)	100.0	0.0	0.0
I felt good in the group (*n* = 77)	98.7	1.3	0.0

^1^ Number of participants out of the 86 intervention participants that completed this question.

## Supplementary Materials

The following are available online at http://www.mdpi.com/1660-4601/15/10/2107/s1, Supplementary File 1: Performance objectives (PO), change objectives (CO), and determinants for promoting mental health among employees in social enterprises in Flanders (Belgium); Supplementary File 2: Theory-based methods and practical strategies to promote mental health among employees in social enterprises in Flanders (Belgium); Supplementary File 3: Data overview.
