# Supplementary material for: Evaluation of a Brief Intervention for Promoting Mental Health among Employees in Social Enterprises: A Cluster Randomized Controlled Trial
Source: Int J Environ Res Public Health. 2018 Sep 25;15(10):2107. doi: 10.3390/ijerph15102107 (PMC6210353; doi:10.3390/ijerph15102107)
Supplement: Supplementary file 1 [file ijerph-15-02107-s001.zip › ijerph-334124-SI/ijerph-334124-supplementary flie 1.pdf]

International Journal of
Environmental Research and Public Health

MDPI

Supplementary File 1: Performance objectives (PO), change objectives (CO) and determinants for promoting mental health among employees in social economy companies in Flanders (Belgium)

**Performance objectives (PO)**	**Determinants**				
	Knowledge = understanding the aim and the content of the intervention	Attitude = a positive or negative evaluation of performing the behaviour	Awareness = need recognition or (problem)solving appraisal	Self-efficacy = subjective probability that the person is capable of executing the behaviour	Perceived social support = support that the person experiences from the social environment to perform the behaviour
PO_0: Decide to participate in the mental health sessions	CO_1: Explain that the intervention consists of talk sessions<br>CO_2: Explain that we will work on the enhancement of a good feeling	CO_3: Express positive feelings about participating in the talk sessions	CO_4: Realize that you can create a good feeling	CO_5: Express confidence that they can participate in talk sessions	
PO_1: Make an assessment of their burden and resilience	CO_6: Explain what burden is<br>CO_7: Explain what resilience is<br>CO_8: Explain that it is difficult to work on a good feeling when you are trying to survive		CO_9: Express their burden and resilience		CO_{10}: Feel recognition for their burden and resilience
PO_2: Searching for help and support	CO_{11}: Explain that they can react to burden by searching for help and support	CO_{12}: Explain that searching for help and support is necessary to reinforce your resilience	CO_{13}: Realize that they are resilient because they search for help and support in case of problems	CO_{14}: Express confidence that they are able to search for help and support	CO_{15}: Feel stimulated and supported by others to search for help and support

Table 1. Continued.

Performance objectives (PO)	Determinants				
	**Knowledge**	**Attitude**	**Awareness**	**Self-efficacy**	**Perceived social influences**
PO_3: Protecting yourself against an increase in burden	CO_{16}: Explain that they can react to burden by protecting themselves	CO_{17}: Explain that protecting yourself is necessary to reinforce your resilience	CO_{18}: Realize that they are resilient because they protect themselves in case of problems	CO_{19}: Express confidence that they are able to protect themselves	CO_{20}: Feel stimulated and supported by others to protect themselves
PO_4: Accepting yourself	CO_{21}: Explain that they can react to burden by accepting themselves	CO_{22}: Explain that accepting yourself is necessary to reinforce your resilience	CO_{23}: Realize that they are resilient because they accept themselves in case of problems	CO_{24}: Express confidence that they are able to accept themselves	CO_{25}: Feel stimulated and supported by others to love themselves
PO_5: Taking care of yourself	CO_{26}: Explain that they can react to burden by taking care of themselves	CO_{27}: Explain that taking care of yourself is necessary to reinforce your resilience	CO_{28}: Realize that they are resilient because they take care of themselves in case of problems	CO_{29}: Express confidence that they are able to take care of themselves	CO_{30}: Feel stimulated and supported by others to take care of themselves
PO_6: Deal with problems in a positive and healthy way (coping)	CO_{31}: Can list the four coping strategies which can provide a good feeling	CO_{32}: Express the need for the use of coping strategies	CO_{33}: Realize which coping strategies they already use and which coping strategies are missing	CO_{34}: Express confidence that they can perform the four coping strategies	CO_{35}: Feel recognition for their opinion and ideas about their coping strategies
