# Supplementary material for: Evaluation of a Brief Intervention for Promoting Mental Health among Employees in Social Enterprises: A Cluster Randomized Controlled Trial
Source: Int J Environ Res Public Health. 2018 Sep 25;15(10):2107. doi: 10.3390/ijerph15102107 (PMC6210353; doi:10.3390/ijerph15102107)
Supplement: Supplementary file 1 [file ijerph-15-02107-s001.zip › ijerph-334124-SI/ijerph-334124-supplementary flie 2.pdf]

International Journal of
Environmental Research and Public Health

MDPI

Table 2. Theory-based methods and practical strategies to promote mental health among employees in social economy companies in Flanders (Belgium).

Determinant	Change objectives*	Theory-based method◦	Theory	Practical strategy
Knowledge	CO_1	Persuasive communicaton	Persuasion-communication matrix; Elaboration likelihood model; Social cognitive theory; Diffusion of Innovations Theory	Invitation
	CO_2	Using imagery	Theories of information processing	Chair
	CO_6	Advance organizers	Theories of information processing	Assignment burden
		Discussion	Theories of information processing	Assignment burden
	CO_7	Advance organizers	Theories of information processing	Assignment resilience
		Discussion	Theories of information processing	Assignment resilience
		Elaboration	Theories of information processing Elaboration likelihood model	Assignment resilience
	CO_8	Advance organizers	Theories of information processing	Symbol content sessions
		Discussion	Theories of information processing	Symbol content sessions
		Elaboration	Theories of information processing Elaboration likelihood model	Symbol content sessions
	CO_{11}, CO_{16}, CO_{21}, CO_{26}, CO_{31}	Advance organizers	Theories of information processing	Assignment resilience
	CO_{11}, CO_{16}, CO_{21}, CO_{26}, CO_{31}	Discussion	Theories of information processing	Assignment resilience
	CO_{31}	Using imagery	Theories of information processing	Chair
Attitude	CO_3	Arguments	Persuasion-communication matrix; Elaboration likelihood model	Invitation
	CO_{12}, CO_{17}, CO_{22}, CO_{27}	Arguments	Persuasion-communication matrix; Elaboration likelihood model	Assignment resilience
	CO_{12}, CO_{17}, CO_{22}, CO_{27}	Elaboration	Theories of information processing Elaboration likelihood model	Assignment resilience
	CO_{32}	Arguments	Persuasion-communication matrix; Elaboration likelihood model	Individual action plan
		Elaboration	Theories of information processing; Elaboration likelihood model	Individual action plan
		Repeated exposure	Theories of learning	Assignment resilience

* The detailed change objectives can be found in Table 1.

◦The theory-based methods can be found in Bartholomew et al., pages 327-344.

Table 2. Continued.

Determinant	Change objectives *	Theory-based method◦	Theory	Practical strategy
Awareness	CO_{4}	Using imagery	Theories of information processing	
	CO_{9}	Scenario-based risk information	Precaution-Adoption Process Model	Chair
		Self-affirmation task	Protection Motivation Theory	Assignment burden and resilience<br>Assignment burden and resilience<br>Assignment burden and resilience
		Active learning	Persuasion-communication matrix;<br>Elaboration likelihood model;<br>Social cognitive theory	Assignment resilience<br>Assignment resilience
	CO_{13}, CO_{18}, CO_{23}, CO_{28}, CO_{33}	Scenario-based risk information	Precaution-Adoption Process Model	Assignment resilience
		Self-affirmation task	Protection Motivation Theory	
	CO_{13}, CO_{18}, CO_{23}, CO_{28}	Active learning	Persuasion-communication matrix;<br>Elaboration likelihood model;<br>Social cognitive theory	Invitation
Self-efficacy	CO_{5}	Verbal persuasion/ exhortation	Social cognitive theory;<br>Theory of self-regulation	Assignment resilience
	CO_{14}, CO_{19}, CO_{24}, CO_{29}	Verbal persuasion/ exhortation	Social cognitive theory;<br>Theory of self-regulation	Assignment resilience
		Active learning	Persuasion-communication matrix;<br>Elaboration likelihood model;<br>Social cognitive theory	Assignment resilience
		Planning coping responses	Attribution Theory and Relapse Prevention Theory;<br>Theories of Goal Directed Behavior<br>Theories of Self-Regulation	Individual action plan
	CO_{34}	Public commitment	Theories of Automatic Impulsive and Habitual Behavior	
		Goal setting	Goal-Setting Theory;<br>Theory of self-regulation	Individual action plan

* The detailed change objectives can be found in Table 1.

◦The theory-based methods can be found in Bartholomew et al., pages 327-344.

Table 2. Continued.

Determinant	Change objectives*	Theory-based method◦	Theory	Practical strategy
Perceived social influences	CO_{10}	Stimulate communication to mobilize social support	Theory of planned behavior;<br>Theory of reasoned action;<br>Diffusion of Innovations Theory;<br>Theories of Social Networks and Social Support	Talk sessions
		Provide opportunities for social comparison	Social Comparison Theory	Talk sessions
		Individualization		Assignment burden and resilience
	CO_{15}, CO_{20}, CO_{25}, CO_{30}, CO_{35}	Stimulate communication to mobilize social support	Trans-theoretical model<br>Theory of planned behavior;<br>Theory of reasoned action;<br>Diffusion of Innovations Theory;<br>Theories of Social Networks and Social Support	Talk sessions
		Provide opportunities for social comparison	Social Comparison Theory	Talk sessions
		Individualization		
	CO35			

* The detailed change objectives can be found in Table 1.

◦The theory-based methods can be found in Bartholomew et al., pages 327-344.
